# Facial trauma followed by osteomyelitis - Case report

**DOI:** 10.1016/j.ijscr.2020.03.009

**Published:** 2020-03-07

**Authors:** Gabriela Caovilla Felin, Cassian Taparello, Vinicios Fornari, Paulo Mesquita Filho, Júnior Grandii, Letícia Copatti Dogenski, João Paulo De Carli

**Affiliations:** a Department of Oral Surgery, Faculty of Dentistry, University of Passo Fundo, Passo Fundo, RS, Brazil; b Resident in Oral and Maxillofacial Surgery, Faculty of Dentistry, University of Passo Fundo, Passo Fundo, RS, Brazil; c Hospital De Clínicas, Passo Fundo, RS, Brazil; d Dentist at the Faculty of Dentistry of University of Passo Fundo, Passo Fundo, RS, Brazil; e Departments of Oral Medicine and Prosthodontics, Faculty of Dentistry, University of Passo Fundo, Passo Fundo, RS, Brazil

**Keywords:** Osteomyelitis, Facial trauma, Debridement, Case report

## Abstract

**Introduction:**

Osteomyelitis is an inflammatory-infectious state that may involve trabecular bone, cortical bone, bone marrow and periosteum. The source of the infection may be hematogenic, acquired from an adjoining infectious focus or by direct inoculation into the bone. Its treatment involves antibiotic administration and surgery, but its management remains challenging.

**Presentation of case:**

A 76-year-old male patient with a history of car accident 3 months earlier sought hospital care with nasal fracture and loss of substance in the right frontotemporal region, where a pectoral muscle free graft was performed to reconstruct the facial defect. The grafted region had hyperemic edges, necrotic appearance, purulent discharge and bone exposure in the nasal dorsum. The initial diagnostic hypothesis was an infectious process due to graft rejection, with likely evolution to osteomyelitis. The surgical procedure was performed by a multidisciplinary team and the patient received the antibiotic regimen according to the antibiogram, with hospital discharge after 39 days of hospitalization.

**Discussion:**

Treatment of osteomyelitis requires the combination of antimicrobial therapy and surgery. Despite surgical and chemotherapeutic advances, it is a difficult condition to treat and there is no universally accepted protocol for treatment.

**Conclusion:**

Surgical treatment was essential for stabilization of the condition. Due to the complexity of this type of infection in the craniofacial region, planning and execution must be carried out through a multidisciplinary team.

## 1 Introduction

Osteomyelitis is a bone inflammation, usually caused by a bacterial infectious agent [1], which may involve trabecular bone, cortical bone, bone marrow or periosteum [2] causing potential functional impairment or permanent disability [3,4].

Osteomyelitis may develop from an infectious site of the underlying soft tissue secondary to trauma, radiotherapy, pressure ulcer and burns. Signs and symptoms of bone infection are often nonspecific. Low fever or local pain are usually present. Drainage, fistula or abscess may be observed [5].

Osteomyelitis requires a multidisciplinary approach to diagnose, plan and evaluate each patient's circumstances [1], involving laboratory tests, imaging and histopathological examination [1,2,5].

Once the diagnosis is established, treatment will involve surgery and antibiotic therapy [4,5]. Aggressive debridement of all necrotic tissues is essential. Afterwards, it may be necessary to stabilize the bone and provide adequate soft tissue coverage. Reconstruction techniques provide elements to repair bone by adding a vascularized covering that increases the concentration of antibiotics in the bone [5]. However, despite the introduction of new antibiotics and diagnostic modalities, the management of cranial osteomyelitis remains a challenge [6].

This study aimed to report a case of post-traumatic osteomyelitis in a 76-year-old man, presenting clinical and imaging characteristics, as well as to discuss the adequate diagnosis and treatment for the injury in question. This work has been reported in line with the SCARE criteria [7].

## 2 Presentation of case

Male patient, 76 years old man, Caucasian, hypertensive, alcoholic, former smoker and with a history of car accident 3 months earlier, referred to Hospital de Clínicas de Passo Fundo, RS, Brazil, with nasal and right orbital zygomatic complex fracture and loss of substance in the right frontotemporal region, where a pectoral muscle free graft surgical procedure was performed in order to reconstruct the facial defect. The patient reported headache and drainage of purulent discharge in the face, with previous long-term hospitalization.

**Fig. 1:**
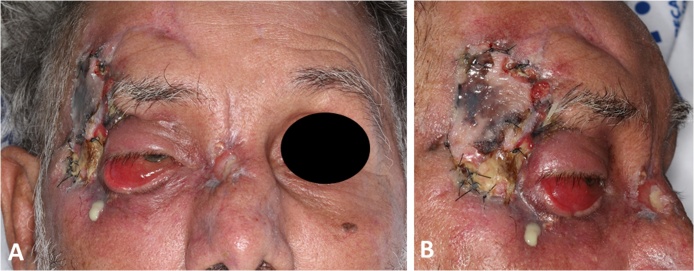
A and B - Initial clinical aspect of the patient showing suppuration, edema and inflammation in the right frontotemporal region, involving the eyeball. Note the presence of suture attaching to the free pectoral graft previously performed.

Physical examination revealed lower eyelid ptosis and right eyelid eversion, right frontotemporal region with graft with hyperemic borders, necrotic appearance and purulent secretion to manual manipulation, with stench and pain on palpation, as well as bone exposure in the back region, on the right side (Fig. 1). The initial diagnostic hypothesis was an infectious process due to the rejection of the free graft performed in the frontotemporal region, with probable evolution to osteomyelitis.

**Fig. 2:**
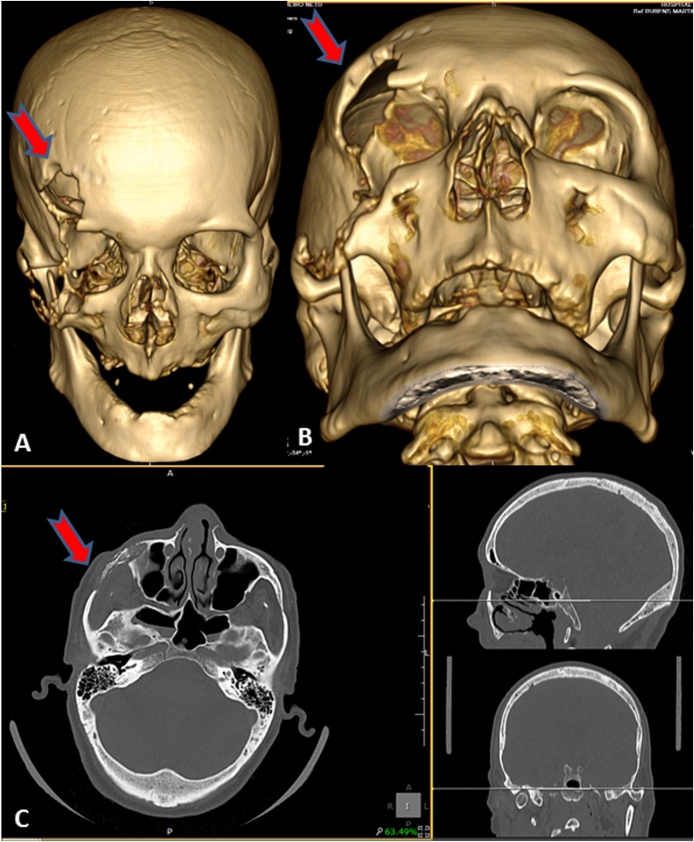
A and B - Computed tomography in three-dimensional reconstruction showing lesion in the right frontotemporal region (red arrows). C - Axial section of tomographic examination indicating destruction of the right orbital cavity (red arrow).

Laboratory tests did not show any particularities. Cranial tomography suggested loss of continuity of the internal bone table along the dura-mater in the frontal and right temporal region, with the aspect of bone sequestrum, besides fracture of the bones of the nose, confirming the clinical suspicion of craniofacial osteomyelitis (Fig. 2). After evaluation of the infectious disease team, the antimicrobial scheme employed was Vancomycin, Cefepime and Clindamycin.

**Fig. 3:**
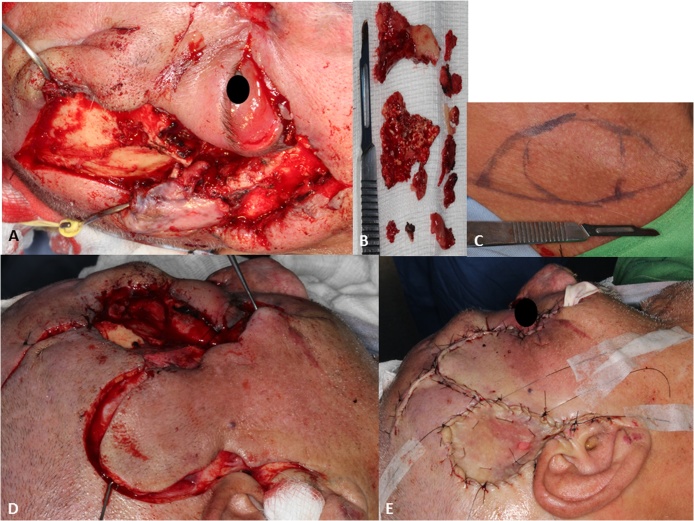
A - Exposure of the surgical. B - Bone sequestrum removed and sent for culture and antibiogram. C - Planning for graft removal in supraclavicular area. D - Operated region after bone sequestrum removal. E - Graft positioned and suture performed.

The surgical procedure involved a multidisciplinary team of oral and maxillofacial surgery, neurosurgery and plastic surgery, and was performed under general anesthesia. Debridement of osteomyelitis was performed, with craniofacial osteotomy in the frontotemporal region and right zygomatic bone, temporal flap rotation in order to cover the defect created after bone curettage and supraclavicular free graft to correct the defect created in the temporal flap rotation region. Necrotic bone was sent for culture and antibiogram tests (Fig. 3).

**Fig. 4:**
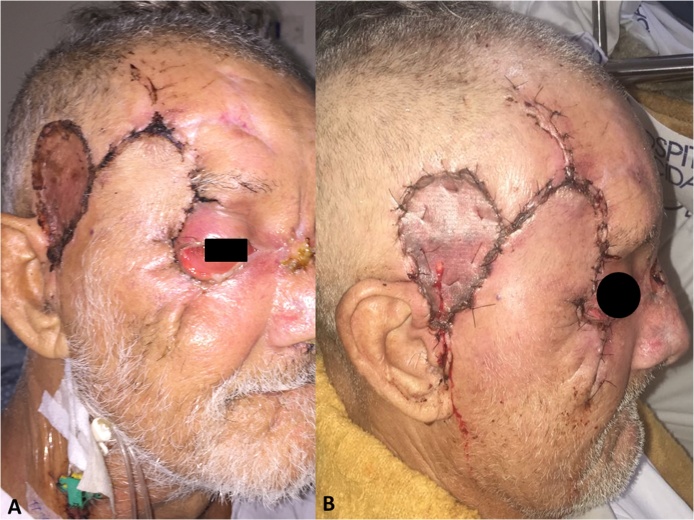
A - Five days postoperative clinical aspect. B - Seven days postoperative clinical aspect.

The patient presented satisfactory postoperative response (Fig. 4). Dressing and drainage were maintained in the frontotemporal graft region for 05 days and the eye occluded for 48 h. Four days after surgery, culture tests showed the presence of *Enterobacter cloacae* sensitive to Amikacin, Polymyxin B, Imipenem and Meropenem. The antimicrobial scheme was adjusted for Meropenem.

**Fig. 5:**
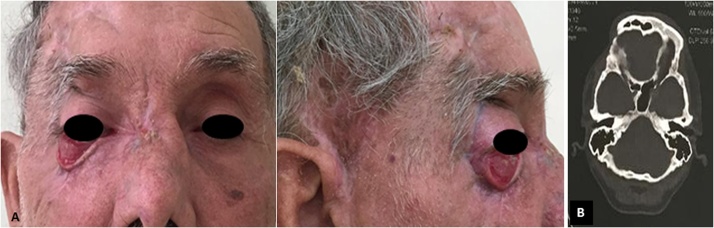
A - Clinical aspect of the patient 12 months after surgery. B - Tomographic appearance 12 months after surgery.

The patient was discharged after 39 days of hospitalization, presenting adequate evolution, no pain symptoms nor new signs of infection, well-preserved operative wounds, viable frontotemporal skin graft with good perfusion, with visual acuity and preserved ocular motricity, but with aesthetic deficit in the right lower eyelid. Outpatient follow-up was performed after 15, 30, 60 and 360 days. During all returns, the patient's only complaint was aesthetic due to the right lower eyelid ptosis (Fig. 5A). Skull tomography performed 12 months after hospital discharge showed no particularity, and there were no new osteolytic areas (Fig. 5B). The patient was instructed to return annually for postoperative control.

## 3 Discussion

The incidence of osteomyelitis is three times higher among individuals aged sixty years old or older. Older people are prone to osteomyelitis because they experience a higher frequency of disorders that lead to infection, such as trauma [8,9]. This statement is in line with the present study, whose patient was 76 years old, with a previous history of trauma and pectoral muscle free graft for facial defect reconstruction.

Retaining osteomyelitis is challenging when the integrity of the surrounding soft tissues is low [4,9]. In this case, the infectious process due to free graft rejection in the frontotemporal region was crucial for the evolution of osteomyelitis.

Clinical suspicion of osteomyelitis in most cases derives from the presence of signs and symptoms of infection, but other diagnostic tools are needed for confirmation. At the onset of the disease, imaging may not present changes. As the disease progresses, periosteal elevation, cortical rupture, spinal cord involvement and osteolysis develop [2,4,10]. In cases where clinical history and imaging are typical, diagnosis can usually be made without the need for biopsy, although in some cases biopsy may be required to exclude malignancy, histiocytosis or other diagnoses [10]. In the case reported, the tomography suggested the presence of loss of continuity of the internal bone table next to the dura-mater in the frontal and right temporal region, with bone sequestrum aspect, confirming, along with the clinical history, craniofacial osteomyelitis.

Optimal treatment of osteomyelitis requires the combination of 6-week antimicrobial therapy and surgery. Despite surgical and chemotherapeutic advances, osteomyelitis remains difficult to treat and there is no universally accepted protocol for treatment [11]. Antibiotic treatment should be based on meticulous cultures performed during debridement surgery or bone biopsies [4,5]. In this case, culture tests showed the presence of *E. cloacae*. Although such bacterium is a common commensal in the gastrointestinal and genitourinary tract, it also exists in environmental samples, including plant matter, soil and water sources. *E. cloacae* has been described as a prominent cause of trauma-associated infection, reflecting its presence in the environment [12].

Surgical treatment involves debridement, removal of necrotic bone and tissue culture, control of dead space and maintenance of bone stability [6,13,14]. This statement confirms the surgical treatment performed on the patient of the clinical case in question.

Bone instability or coverage defects as a result of debridement also need attention, as reconstruction of bone and soft tissue defects may influence the outcome. Reconstruction techniques provide elements for bone repair by adding a vascularized covering that increases the concentration of antibiotics in the bone and aids in its healing [5]. In this case, the patient received a supraclavicular free graft in order to correct the frontotemporal defect, which was viable during the follow-up period, presenting good perfusion.

## 4 Conclusion

Based on the treatment of the described clinical case, it is noted that osteomyelitis is a very complex infection and may settle after traumatic episodes involving the craniofacial region. The surgical treatment and the correct antibiotic regimen instituted by a multidisc ciplinary team proved to be essential for the stabilization of the condition.

## Sources of funding

The authors state that the present study had no sponsor or source of funding.

## Ethical approval

Because this is a case report, the present study was not appreciated by a research ethics committee. However, it follows as an attached file a patient's fully informed written consent for publication of the reported clinical case.

## Consent

Written informed consent was obtained from the patient for publication of this case report and accompanying images. A copy of the written consent is available for review by the Editor-in-Chief of this journal on request.

## Author contribution

Gabriela Caovilla Felin – Execution of the surgical step; acquisition of data.

Cassian Taparello – Analysis and interpretation of imaging exams; analysis and interpretation of data.

Vinicios Fornari – Execution of the surgical step; acquisition of data.

Paulo Mesquita Filho and Júnior Grandii – Execution of the surgical step; acquisition of data. Letícia Copatti Dogenski – Literature review, translation and spelling revision; conception and design of the study.

João Paulo De Carli – Writing work, discussion and final approval; conception and design of the study.

## Registration of research studies

The present study is not a research involving humans, but a clinical case report, whose patient authorized the publication by means of a free and informed consent term.

## Guarantor

João Paulo De Carli.

## Provenance and peer review

Not commissioned, externally peer-reviewed.

## Declaration of Competing Interest

None of the authors has any conflict of interest.
